# The effect of deletion of the orphan G – protein coupled receptor (GPCR) gene *MrgE *on pain-like behaviours in mice

**DOI:** 10.1186/1744-8069-4-2

**Published:** 2008-01-15

**Authors:** Peter J Cox, Tom Pitcher, Steven A Trim, Christine H Bell, Wenning Qin, Ross A Kinloch

**Affiliations:** 1 Pain Therapeutics, Discovery Biology, Pfizer Global Research and Development, Sandwich, Kent CT13 9NJ, UK; 2 Genetically Modified Models Centre of Emphasis, Pfizer Global Research and Development, MS 8118D-3110, Eastern Point Road, Groton, CT 06340, USA

## Abstract

**Background:**

The orphan GPCR *MrgE *is one of an extended family of GPCRs that are expressed in dorsal root ganglia (DRG). Based on these expression patterns it has been suggested that GPCRs like *MrgE *may play a role in nociception however, to date, no direct supporting evidence has emerged. We generated mutant mice lacking *MrgE *and examined the effects of deletion of this gene in three pain behavioural models. The effect of *MrgE *gene deletion on expression of *Mrgs *and genes involved in sensory neurone function was also investigated.

**Results:**

The absence of *MrgE *had no effect on the development of pain responses to a noxious chemical stimulus or an acute thermal stimulus. However, in contrast, the development but not the maintenance of neuropathic pain was affected by deletion of *MrgE*. The expression of *Mrg *genes was not significantly affected in the *MrgE *knockout (KO) mice with the sole exception of *MrgF*. In addition, the expression of 77 of 84 genes involved in sensory neuron development and function was also unaffected by deletion of *MrgE*. Of the 7 genes affected by *MrgE *deletion, 4 have previously been implicated in nociception.

**Conclusion:**

The data suggests that *MrgE *may play a role in selective pain behavioural responses in mice.

## Background

Mrg-receptors, first described in 2001 [1], constitute a family of G-protein coupled receptors in which certain members are expressed in distinct subsets of sensory neurons, known to play a role in nociception, and were activated by peptides that had previously been functionally linked to pain. Indirect physiological evidence for a role for *MrgC*, in particular, was provided by the demonstration that peptides known to activate *MrgC *elicited pain-like behaviours on administration to rats [2]. Recently it has been shown that levels of one of these peptides, Bovine Adrenal Medulla peptide 22, was increased in superficial laminae of the spinal cord (SC) and in DRG cells following complete freunds adjuvant induced inflammation [3]. However the specificity of these peptides for *MrgC *has not been conclusively demonstrated, therefore non-MrgC mediated effects cannot be ruled out. Expression of rat *MrgC *and *MrgA *is down regulated in DRG following spinal nerve ligation provided additional evidence that these receptors are somehow functionally linked to pain [4]. Elegant studies with genetically modified mice demonstrated that *MrgD *is uniquely expressed in primary afferent nerve fibres but pain behavioural phenotypes for mice lacking this receptor were not reported [5]. The MrgD ligand, beta-alanine, inhibited potassium channel mediated M-current activity in both a recombinant cell system and primary DRG neurons suggesting that activation of MrgD could inhibit the M-current resulting in repetitive firing of normally phasic nociceptive neurons leading to pain [6]. Similarly activation of MrgX1 expressed in rat superior cervical ganglion neurons resulted in M-current inhibition [7].

A functional role for Mrg receptors in other systems could be assumed from the physiological functions of ligands known to activate these receptors. MrgX2 is activated by basic molecules [8] and cortistatin [9], amongst other ligands, and is therefore postulated to have a role in mast cell degranulation and neuroendocrine function respectively. Murine MrgA1 and MrgC11 are activated by RF-amide peptides [10] suggesting that these receptors could be involved in a variety of neuromodulatory functions attributed to these peptides [11]. However, as with pain, direct physiological proof for a role for these receptors in these systems is lacking.

Study of members of this family is generally hampered by the lack of specific ligands and/or pharmacological tools. *MrgE *is an orphan receptor; however we and others have demonstrated that this receptor is largely restricted to the nervous system with greatest expression in SC and DRG [12]. In the absence of suitable tools we have developed genetically modified mice lacking *MrgE*. The phenotype of *MrgE *KO mice was studied in a number of assays, with particular focus on pain behaviours. The effect of deletion of *MrgE *on expression of a selection of genes known to be expressed in sensory neurons, including other *Mrgs*, was also investigated.

## Results

### Generation of *MrgE *KO mice

**Figure 1:**
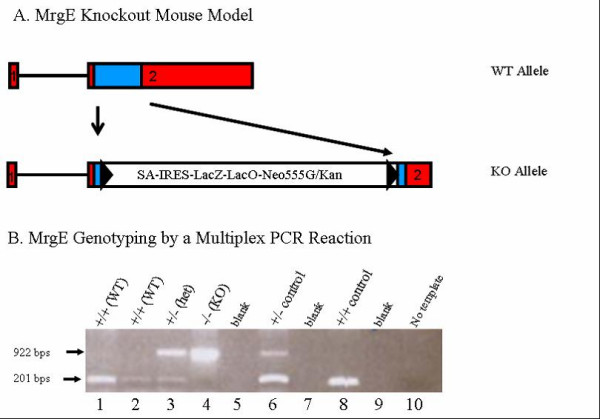
**Gene targeting strategy for generation of *MrgE *KO mice**. (A) The targeting vector was assembled on the pGT-N28 backbone (NEB, Ipswich, MA) and so designed such that a total of 776 base pairs from within the 930 base pair coding region located in exon 2 of the 2 exon *MrgE *gene (first exon is un-translated) was deleted and replaced with a 7,076 base pair IRES-lacZ reporter and neomycin resistance cassette. Solid Line: intron 1; boxes colored red: exons 1 and 2; box colored blue: coding sequence located in exon 2; uncoloured box: IRES-LacZ-NEO cassette replacing part of the coding sequence located in exon 2; black triangle: FRT sequence. (B) Genotyping assay was designed to differentiate among wild type (+/+), heterozygote (+/-) or homozygote knockout (-/-) *MrgE *mice and performed using a multiplex PCR reaction, with a shared 5' forward primer (5'-GCA GAC ATC AGC CAT GAC GT-3') and a 3' reverse primer unique to the targeted allele (#2416, 5'-ATC AGC TTA CCA TGG CCA AGA TCC C-3') or to the *MrgE *locus (5'-ATC TAT CTC TTG GAT GTG GCC TG-3'). The WT amplicon is 201 and KO allele 922 bps.

**Figure 2:**
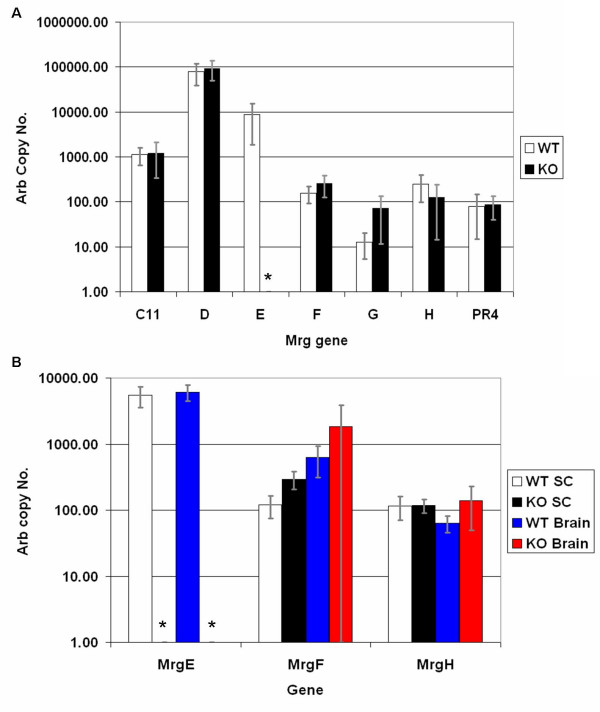
**Quantitative RTPCR analysis of *Mrg *expression**. Quantitative RTPCR analysis of *Mrg *expression in (A) DRG and (B) SC and brain from WT and KO mice. The asterisks indicate that no copies of *MrgE *were detected.

*MrgE *KO mice were generated using a standard homologous recombination strategy. Southern blot confirmed homologous recombination had occurred (data not shown) and a PCR genotyping strategy was used to genotype mouse tail DNA samples (see Fig. 1). The genotype of the *MrgE *KO animals was confirmed by absence of detectable *MrgE *transcripts in all tissues tested. TaqMan quantitative PCR confirmed the presence of *MrgE *expression in DRG, SC and brain from wild-type (WT) mice; whereas *MrgE *expression was not detected in homozygote *MrgE *KO mice (see Fig. 2).

### Behavioural testing of KO and WT mice

KO and WT mice were subjected to a battery of behavioural and biochemical tests. KO mice appeared to be normal in the majority of tests with some minor differences noted in blood biochemistry, sexual behaviour and prepulse startle responses. However no overt phenotypic abnormalities were identified (Data not shown).

**Figure 3:**
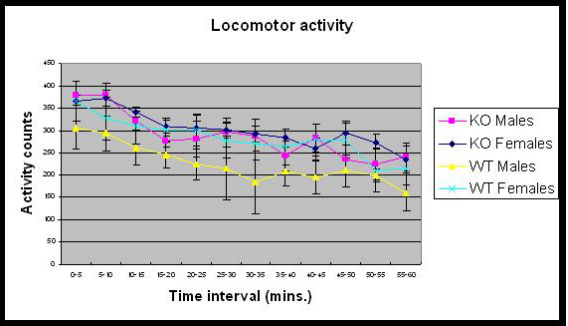
**Locomotor activity of WT and KO mice**. Data is Mean ± SEM of 3–4 mice per group expressed as activity counts per 5 minute bin intervals for 1 hour. No significant effect was seen between the groups over the time course.

The locomotor activity of mice was assessed demonstrating no difference in the general motor coordination of WT and KO mice (see Fig. 3).

**Figure 4:**
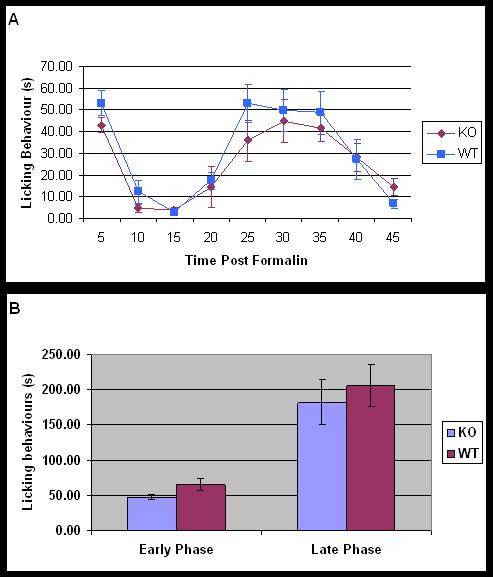
**Formalin test of WT and *MrgE *KO mice**. (A) The time course of development of formalin responses in KO and WT mice. (B) The early and late phase of the formalin response in KO and WT mice showing a trend towards reduction in early phase behaviours in KO mice.

Intraplantar injection of formalin resulted in a biphasic nocifensive response in both WT and *MrgE *KO mice (see Fig. 4). There was a trend towards reduced nocifensive behaviours in the KO mice in the first phase of the response to formalin (see Fig. 4C); however this did not reach statistical significance. Overall, no significant difference in the frequency or timing of nocifensive behaviours between WT and KO mice was detected in either the first or second phase of the formalin test. Likewise, hotplate responses of WT and KO mice were similar (data not shown)

**Figure 5:**
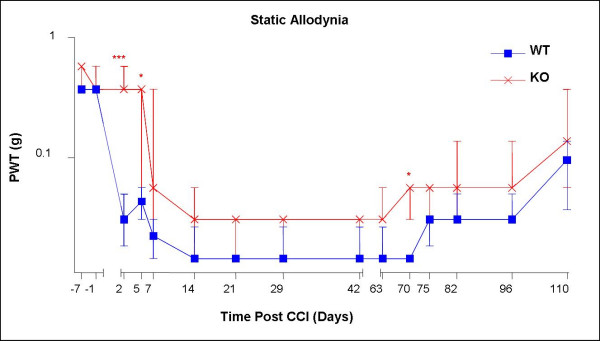
**Chronic constriction injury of WT and *MrgE *KO mice**. The punctuate allodynia data are expressed as median force (g) required to induce a PWT in wild-type (n = 13) and KO (n = 6) mice (vertical bars represent the first and third quartiles). ***P < 0.01, *P < 0.05 unpaired T Test comparing WT and KO mice at each time point.

KO and WT mice developed ipsilateral punctate allodynia after chronic constriction injury (CCI) of the sciatic nerve. A statistically significant difference in the rate of development of allodynia was demonstrated between WT and *MrgE *KO mice (see Fig. 5). The onset of maximal allodynia was delayed in the KO in comparison to the WT mice, with the most significant difference in allodynia noted in the day 2 to 7 period post surgery. Otherwise, the WT and *MrgE *KO mice maintained a similar level of allodynia indicated by a paw withdrawal threshold of 0.04 g or less. The onset of recovery at approximately 100 days post surgery was also similar in both groups.

### Gene expression analysis

**Figure 6:**
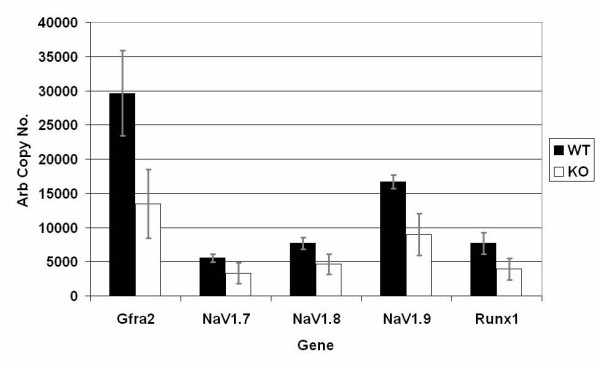
**Quantitative RTPCR analysis of DRG expression of genes involved in sensory neuron function in WT and KO mice**. RT2 arrays revealed several sensory neurone genes were reduced in DRG of the KO relative to WT animals (p = <0.05, p = *0.07). Error bars represent standard deviation.

The expression of *Mrg *gene family members together with genes important for sensory neuron function and development were examined in various tissues from adult WT and *MrgE *KO mice by quantitative RTPCR analysis. In WT animals, *MrgD *and *E *are the most abundant *Mrgs *in DRG (see Fig. 2A); whereas *MrgE *is the most abundant *Mrg *in SC and brain with 6000 and 3000 arbitrary copies respectively (see Fig. 2B). There is no statistical difference in the expression of *MrgC11*, *D*, *F*, *G*, *H *and *PR4 *genes in DRG of KO and WT mice. However, *MrgF *is up-regulated 2-fold (p = 0.0013) in the SC of KO animals (see Fig. 2B). *MrgC11 *and *D *expression was detected at extremely low levels in brain and SC preparations from some, but not all, mice while *MrgPRa4 *or *MrgG *expression was not detected in brain or SC (data not shown). Use of the RT^2 ^PCR profiler array (SuperArray) consisting of genes implicated in patterning, development and/or function of sensory neurones revealed a trend towards reduced expression of *GFR α-2*, *Na*_*v*_*1.7*, *Na*_*v*_*1.8*, and *Runx-1 *in DRG from KO versus WT mice (see Fig. 6) (p = <0.05, p = *0.07).

**Figure 7:**
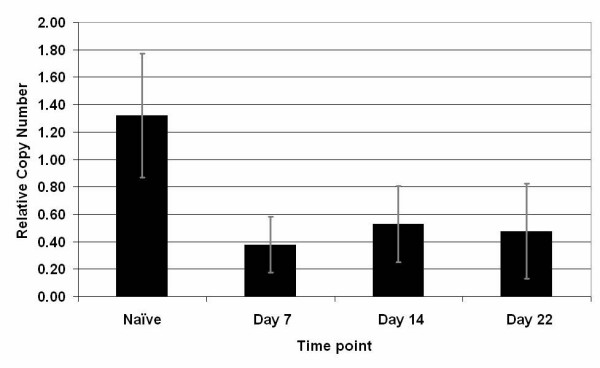
**Quantitative RTPCR analysis of *MrgE *expression post CCI in the rat**. *MrgE *expression was reduced in ipsi-versus contralateral sciatic nerve from rats 7, 14 and 21 days post CCI surgery (n = 6 per time point) (Error bars represent standard deviation P < 0.01). Expression of *MrgE *in ipsi- and contralateral sciatic nerve from naïve animals was unchanged (data not shown).

The effect of chronic constriction injury of the sciatic nerve on *MrgE *gene expression was examined in the rat. Expression of *MrgE *in the injured sciatic nerve relative to the uninjured sciatic nerve was significantly reduced at all time points post surgery tested (see Fig. 7).

## Discussion

*MrgE *KO and WT mice are indistinguishable in the formalin and hotplate tests implying that *MrgE *plays no significant role in the response to noxious chemical and thermal stimuli respectively. Dong *et al *2001 demonstrated that *MrgD *and *MrgA1 *are expressed in vanniloid receptor-1 (VR1) negative small diameter sensory neurones in the mouse, indicating that these receptors may be less important for the detection of noxious chemical stimuli. At present the detailed distribution of other Mrgs, including *MrgE*, in rodent DRG has not been determined. Taken together, the KO and distribution data would suggest that *Mrgs*, in general, may not be involved in the sensing of chemically induced pain, although this may not be the case for higher mammals where distribution of *MrgD *and *E *in VR1 positive and medium to large diameter neurones, respectively, was detected in DRG [13].

There was a significant difference between WT and KO mice in the rate they develop an increased hypersensitivity to the application of von Frey hairs. However the peak level of allodynia acquired, and maintained, was similar in both WT and KO mice. The slower rate of onset of allodynia in the KO animals suggests that *MrgE *plays a role in the development but not the maintenance of allodynia. Whether this behavioural effect is directly due to lack of functional *MrgE *in adult mice or has arisen due to the absence of *MrgE *during the development of the nervous system in KO animals remains to be seen. In the adult rat *MrgE *expression is significantly reduced as early as day 7 post CCI of the sciatic nerve lending further support to the notion that this gene may be involved in the development of allodynia. Down regulation of *MrgE *or a reduction in cells expressing *MrgE *may be an essential component of the temporal processes that affect the development of allodynia, therefore the complete absence of this gene deregulates these processes slowing the development of allodynia in the KO mice.

Emerging data and hypotheses suggest that cellular and functional plasticity involving neuro-immune interaction and neuronal long term facilitation, potentiation and depression in the periphery, at spinal and/or supraspinal sites may underlie behaviours such as allodynia associated with persistent pain states (For recent reviews see [14,15]). Although MrgE is expressed at all tissue levels known to be involved in pain sensation, the KO data suggests that it has no role in maintaining the long term plastic changes that may underlie neuropathic pain. This notion is supported by the observation that KO and WT animals develop the same degree of allodynia.

The small but statistically significant down regulation of genes known to be involved in sensory neuron function and development is of interest and may also offer an explanation. It is tempting to speculate that reduction in the expression of these genes observed in the DRG of KO mice may reflect a reduction in the number of neurons that express these same genes. The loss of these sensory neurons may result in the observed deficit in the rate of development of allodynia. Deficits in pain-like behaviours have been demonstrated in *GFR α-2 *[16]*Na*_*v*_*1.7 *[17]*Na*_*v*_*1.8 *[18,19] and *Runx-1 *[20] KO mice, although the extent and nature of these behavioural deficits are much more dramatic than those demonstrated with the *MrgE *KOs, possibly because of complete loss of expression of the genes under study.

The expression of only one *Mrg*, *MrgF *was shown to be altered by *MrgE *deletion. *MrgD *gene expression was unaffected by *MrgE *deletion although both these receptors have been shown to functionally associate in a recombinant cell system [12]. Although speculative, upregulation of *MrgF *may compensate for the absence of *MrgE *therefore it would be interesting to determine if co-expression of *MrgF *had any effect on *MrgD *function, or if the pain behavioural phenotype of an *MrgF MrgE *double KO would be more profound than that observed with *MrgE *KOs.

## Conclusion

The data presented in this paper is the first to demonstrate a role for the orphan GPCR *MrgE *in pain. The functional significance of *MrgE *deletion on pain will require extension of these studies to detailed molecular and immunohistochemical analysis of the KO mice, and further analysis of the effects of *MrgE *gene deletion on other pain endpoints and in other pain models.

## Methods

### Generation of *MrgE *KO mice

*MrgE *targeted ES clone was obtained from Deltagen (San Carlos, CA.). A genomic fragment containing the *MrgE *gene was isolated from a mouse genomic phage library. The targeting vector was assembled on the pGT-N28 backbone (NEB, Ipswich, MA) and so designed that a total of 776 bps from within the 930 base pair coding region located in exon 2 of *MrgE *gene (first exon is un-translated) was deleted and replaced with a 7,076 bp IRES-lacZ reporter and neomycin resistance cassette (IRES-LacZ-NEO) (see Fig. 1A). The targeting vector was linearized and electroporated into the E14 (129/Ola) mouse embryonic stem (ES) cells. ES cells were then cultured in the presence of the antibiotic G418/geneticin, and surviving colonies carrying the homologously integrated neo DNA were identified by PCR amplification, retrieving a 6.5 kb fragment, using a neo-specific reverse primer (#2416, 5'-ATCAGCTTACCATGGCCAAGATCCC-3') paired with a forward primer located 5' to the 5' homology arm (#71732, 5'-GACATCTCCCTAGTCCAGACGACTC-3'). Successful homologous recombination at the 3' end was also confirmed using a neo-specific forward primer (#1431, 5'-ACGTACTCGGATGGAAGCCGGTCTT-3') paired with a reverse primer located 3' to the 3' homology arm (#71738: 5'-CCTCCTTCTCTCTCCACGTGTTCCT-3'), retrieving a 7.0 kb fragment. Colonies that gave rise to the correct PCR products were confirmed by Southern blot analysis and presence of a single neo cassette in the genome was also confirmed by Southern blot analysis using a neo gene fragment as a probe (data not shown).

Targeted ES cells were injected into C57Bl/6J blastocysts and male chimeric mice were generated and bred with C57Bl/6J female mice to produce F1 heterozygous offspring. F1 germline mice were then bred to C57BL/6J wild type mice to expand the colony and heterozygote mice were interbred to produce the study population mice. All phenotypic analysis was performed on a hybrid C57Bl/6J/129 background. Genotyping to detect wild type, heterozygote and homozygote mice was performed using a multiplex PCR reaction, with a shared 5' forward primer (5'-GCA GAC ATC AGC CAT GAC GT-3') and a 3' reverse primer unique to the targeted allele (#2416, 5'-ATC AGC TTA CCA TGG CCA AGA TCC C-3') or to the *MrgE *locus (5'-ATC TAT CTC TTG GAT GTG GCC TG-3'). The WT product is 201 and mutant allele 922 bps (see Fig. 1B).

### Behavioural testing of KO and WT mice

All animals were handled in accordance with Pfizer policy on animal welfare and with regional government legislation on the use of animals in research.

The spontaneous locomotor activity of the mice was measured by placing the animals in a novel environment which is monitored for 1 hr in a 35 × 20 cm perspex chamber equipped with a series of two photocells located at 1 and 10 cm above the floor. Each animal was placed in the centre of the cage and total locomotor activity (ambulation and rearing) was monitored every 5 min and recorded as activity counts as the beams were broken. Activity data was captured on computer. Mice were randomly placed in the photo beam equipped cages.

Acute pain responses were assessed using the formalin and hotplate tests. Mice were subjected to the formalin test as follows. Homozygote KO mice and WT littermates were habituated to perspex boxes for 15 minutes before 20 ul of 5% formalin was administered into the plantar surface of the right hind paw. Nocifensive behaviour was assessed by measuring the time spent licking and biting in 5-minute bins for 45 minutes following formalin injection.

Neuropathic pain behaviours in mice were assessed following CCI of the right sciatic nerve based on previously described methods [21]. Mice were placed in an anaesthetic chamber and anaesthetised with a 2% isofluorane O_2 _mixture. The right hind thigh was shaved and swabbed with 1% iodine. Animals were then transferred to a homeothermic blanket for the duration of the procedure and anaesthesia maintained during surgery via a nose cone. The skin was cut along the line of the thighbone and the common sciatic nerve was exposed at the middle of the thigh by blunt dissection through biceps femoris. Three ligatures (4-0 silk) were tied loosely around the nerve, the wound was closed and the animals allowed to recover. Following surgery the development of static allodynia was monitored regularly over a period of 4 weeks. Static allodynia was evaluated by application of von Frey hairs (Stoelting, Wood Dale, Illinois, U.S.A) in ascending order of force (0.008, 0.02, 0.04, 0.07, 0.16, 0.4, 0.6, 1, 1.4, 2, 4 grams) to the plantar surface of hind paws. Each von Frey hair was applied to the paw for a maximum of 6 seconds, or until a withdrawal response occurred. Once a withdrawal response was established, the paw was re-tested, starting with the filament below. The lowest amount of force required to elicit a response was recorded as paw withdrawal threshold (PWT) in grams. Static allodynia was defined as present if animals responded to a force equal to or less than, 0.04 g, which is innocuous in normal mice. CCI of the rat sciatic nerve was performed as described previously [21].

### Gene expression analysis

*Mrg *family members and genes important for sensory neuron development and function were quantified in various tissues from adult WT and *MrgE *KO mice by quantitative RTPCR analysis. DRG, SC and brain tissues (n = 6) were harvested from KO and WT mice, stored in RNAlater (Ambion) at -20°C. Tissues were mechanically homogenised using Powergen 125 (Fisher) in 600 ul Buffer RLT (Qiagen) containing β-Mercaptoethanol (10 ul/ml RLT). SCs and brains were homogenised whole in larger volumes (1.2 ml and 6 ml) accordingly and RNA was extracted as per the RNeasy tissue protocol (Qiagen) with on column DNaseI treatment. Brains and SCs were also treated with Amplification Grade DNaseI (Invitrogen) in accordance with the manufacturer's instructions. RNA was reverse transcribed using the GeneAmp RNA PCR kit (Applied Biosystems) and 15 ng/well equivalent of cDNA used in quantitative PCR TaqMan^® ^to determine gene expression levels of *MrgC11*, *D*, *E *and *G *using custom oligos (Sigma) and *MrgF*, *H *and *PR4 *using Assays on Demand™ (Applied Biosystems). Arbitrary copy number (ACN) was calculated using the formula ACN = 10^(12-(0.3xCt))^. Multiple gene profiling of the DRGs was performed using 1 ng cDNA per well on a custom RT^2 ^PCR profiler array (SuperArray) and the data analysed using Spotfire Decision site 8.1. The ACN +/-standard deviation was calculated as described. Sciatic nerve was harvested from rats 7, 14 and 21 days post CCI. RNA was isolated from sciatic nerve ipsilateral and contralateral to the injured nerve. cDNA was synthesized from isolated sciatic nerve RNA as described for murine RNA. 25 ng/well of cDNA equivalent was used as template in rat *MrgE *specific TaqMan assays and the data generated analysed using a ΔΔCt method (Applied Biosystems). Ipsilateral versus contralateral levels of *MrgE *were compared for each time point post surgery and expressed as relative copy number. The sequences of all primers and probes used in this study are available on request.

## Competing interests

All authors are employees of Pfizer Global Research and Development and may own/have stock options for Pfizer Inc.

## Authors' contributions

TP and CHB performed the pain behavioural tests. SAT performed gene expression studies. WQ was involved in coordination of production and genotyping of KO animals. PJC and RAK conceived of the study, and participated in its design and coordination, and drafted the manuscript. All authors read and approved the final manuscript.
